# Redox biology underlying the efficacy and toxicity of colistin as a last-resort antibiotic

**DOI:** 10.3389/fmicb.2026.1953981

**Published:** 2026-09-03

**Authors:** Ricardo Jiovanni Soria-Herrera, Walter Ángel Trujillo-Rangel, Leonel García-Benavides, Elodia Nataly Diaz-De la Cruz

**Affiliations:** 1 Facultad de Químico-Farmacobiología, Universidad Michoacana de San Nicolás de Hidalgo, Morelia, Mexico; 2 Centro Universitario de Tonalá, Universidad de Guadalajara, Guadalajara, Mexico

**Keywords:** antioxidants, colistin, last-resort antibiotics, nephrotoxicity, redox biology, redox modulation

## Abstract

The increasing prevalence of multidrug-resistant Gram-negative bacteria has reinforced the clinical importance of last-resort antibiotics such as colistin. Their antibacterial efficacy has made them indispensable for treating life-threatening infections, yet their clinical use is constrained by substantial toxicity. Increasing evidence indicates that redox biology may influence both the antibacterial activity and host toxicity of colistin through distinct biological processes. Reactive oxygen species contribute to bacterial killing while simultaneously promoting mitochondrial dysfunction, oxidative stress, inflammation, and tubular cell death within the kidney. This mini review summarizes current knowledge on the redox mechanisms underlying the antibacterial activity and host toxicity of last-resort antibiotics, with particular emphasis on colistin-induced nephrotoxicity. We discuss experimental and clinical evidence supporting host-directed redox modulation as a strategy to mitigate renal injury, highlighting the limitations that have hindered successful clinical translation. Finally, we examine emerging opportunities, including biomarker-guided strategies and mitochondria-targeted interventions, that may facilitate the development of safer therapeutic approaches while preserving the antibacterial efficacy of colistin.

## 1 Introduction

Antimicrobial resistance (AMR) represents a critical challenge to global public health, reducing the effectiveness of available antibiotics and increasing morbidity, mortality, and healthcare costs worldwide (Antimicrobial Resistance Collaborators, 2022; Ho et al., 2025; Okeke et al., 2024; Sinha and Upadhyay, 2025). The increasing burden of multidrug-resistant Gram-negative bacteria is particularly concerning, with projections suggesting that AMR could cause up to 10 million deaths annually by 2050 in the absence of effective interventions, although it is important to note that these long-term projections have drawn methodological criticism regarding their modeling assumptions (de Kraker et al., 2016; O’Neill, 2016). These projections emphasize that extending the clinical lifespan of existing antibiotics is now as important as developing new antimicrobial agents. This scenario highlights the need for therapeutic strategies that preserve antimicrobial efficacy while reducing treatment-associated toxicity and improving clinical outcomes (Sinha and Upadhyay, 2025; Tang et al., 2023).

Redox biology plays a central role in the dynamic interactions between bacterial pathogens and the host during infection. Under physiological conditions, reactive oxygen species (ROS) are critical mediators of redox signaling, contributing to immune defense, bacterial adaptive responses, and cellular homeostasis. However, excessive ROS production exceeds the capacity of antioxidant defense systems, disrupting redox balance and resulting in oxidative stress and tissue damage (Reddy, 2023). Both host cells and bacteria possess tightly regulated antioxidant defense systems that maintain intracellular redox balance and protect against oxidative damage (Dagah et al., 2024; Reniere, 2018; Shekhova, 2020). In the context of antimicrobial therapy, antibiotics also perturb redox homeostasis, influencing ROS generation in ways that contribute not only to bacterial killing but also to bacterial adaptive responses and host susceptibility to drug-induced toxicity (Donkor and Dahl, 2026; Zhao et al., 2026).

Last-resort antibiotics illustrate this redox-dependent balance particularly well. Among them, colistin remains one of the most valuable therapeutic options against carbapenem-resistant Gram-negative pathogens (Rhouma et al., 2023). Despite its clinical importance, its use is frequently limited by dose-dependent nephrotoxicity, in which oxidative stress, mitochondrial dysfunction, inflammation, and regulated cell death have been consistently implicated (Jafari and Elyasi, 2021; Zhao et al., 2026). The involvement of redox processes in both bacterial killing and host toxicity highlights the dual role of redox biology during colistin therapy and suggests that redox modulation may help improve the balance between antimicrobial efficacy and host safety.

**FIGURE 1:**
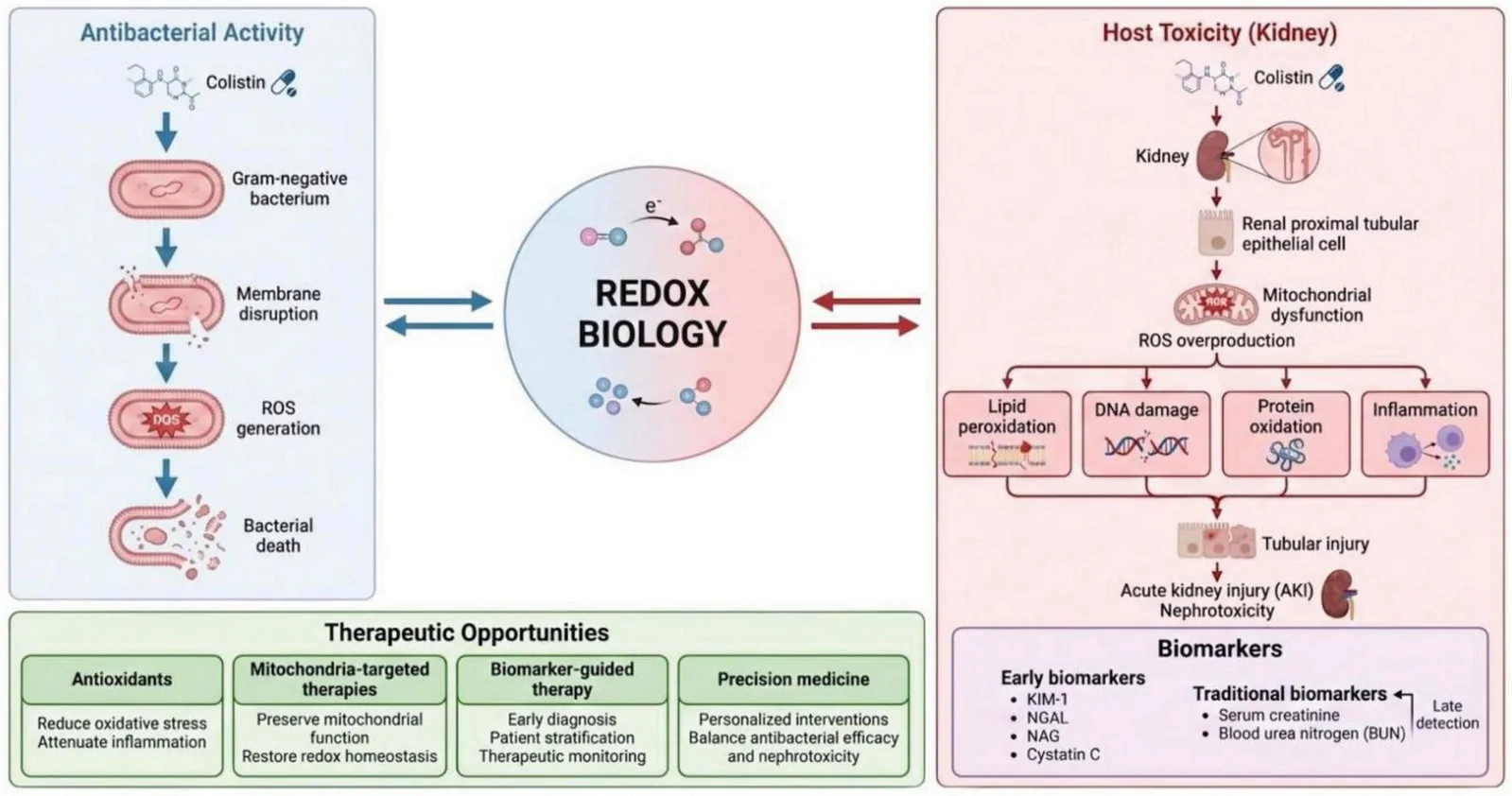
Dual role of redox biology in the efficacy and toxicity of last-resort antibiotics. Schematic overview of the mechanisms linking colistin antibacterial activity with host nephrotoxicity. In bacteria, colistin disrupts the Gram-negative outer membrane and may induce redox alterations associated with bacterial death. In renal proximal tubular epithelial cells, intracellular accumulation promotes mitochondrial dysfunction, oxidative stress, inflammation, and tubular injury, ultimately contributing to nephrotoxicity. Potential redox-based adjuvant strategies aimed at mitigating renal injury while preserving antibacterial efficacy are also illustrated. Created in BioRender. Diaz-De la Cruz, E. N. (2026) https://BioRender.com/yq79swm.

This mini-review reviews the role of redox biology in colistin efficacy and toxicity, highlighting redox modulation as a potential strategy to reduce nephrotoxicity while preserving antibacterial activity. Current knowledge gaps and future translational opportunities are also discussed (Figure 1).

## 2 The therapeutic dilemma of colistin as last-resort antibiotic

Colistin is recognized as an indispensable therapeutic option for the treatment of infections caused by carbapenem-resistant Gram-negative pathogens, particularly *Acinetobacter baumannii*, *Pseudomonas aeruginosa*, and Enterobacterales. As a polymyxin antibiotic, colistin targets lipid A in the outer membrane of Gram-negative bacteria, disrupting membrane integrity and leading to bacterial cell lysis (RoyChowdhury et al., 2025).

The clinical value of colistin is increasingly constrained by the emergence and spread of resistance. Bacteria evade polymyxin activity primarily through lipid A remodeling, which reduces antibiotic binding to the Gram-negative outer membrane. These modifications may result from chromosomal mutations affecting two-component regulatory systems or from the acquisition of plasmid-mediated *mcr* genes, which encode phosphoethanolamine transferases that modify lipid A (Rhouma et al., 2023; Taglialegna, 2023). Together, these resistance mechanisms further restrict the therapeutic utility of colistin, underscoring the need for strategies that preserve its antibacterial activity while limiting dose escalation.

Resistance represents only part of the therapeutic challenge, as the narrow therapeutic window of colistin also limits its safe and effective use. Nephrotoxicity remains the principal dose-limiting adverse effect and represents a major barrier to optimal dosing. The molecular basis of colistin toxicity involves multiple interconnected mechanisms, including intracellular drug accumulation, mitochondrial dysfunction, oxidative stress, inflammatory signaling, and apoptosis (Gai et al., 2019; Jafari and Elyasi, 2021). As a result, attempts to increase colistin exposure to overcome bacterial resistance may also intensify host toxicity, creating a therapeutic dilemma in which optimizing antimicrobial efficacy may increase the risk of treatment-associated toxicity. These limitations have shifted attention toward biological processes that may influence both bacterial responses and host toxicity during antibiotic exposure. Redox biology provides one such framework, as alterations in redox homeostasis have been implicated, although with different levels of evidence, in both the antibacterial effects and tissue toxicity of colistin. Understanding these distinct but interconnected redox responses may facilitate the development of adjunctive interventions aimed at improving the therapeutic balance between antimicrobial efficacy and host safety (Kashi et al., 2026; Mirjalili et al., 2022).

## 3 Redox biology in bacterial killing

During bacterial infection, host and pathogen engage in a dynamic interplay in which redox biology plays a central role. As part of the innate immune response, neutrophils and macrophages rapidly migrate to sites of infection and produce large amounts of ROS and reactive nitrogen species (RNS) through the respiratory burst. These highly reactive molecules contribute directly to bacterial killing by damaging cellular macromolecules. Beyond their direct antimicrobial activity, ROS also serve as signaling molecules that activate NF-κB and other redox-sensitive pathways, promoting inflammasome assembly and the production of proinflammatory cytokines that coordinate antimicrobial defense and immune response (Dagah et al., 2024; Mullen et al., 2020).

To counteract host-derived oxidative stress, bacteria have evolved antioxidant defense systems that preserve intracellular redox homeostasis. Enzymes such as superoxide dismutase, catalase, peroxiredoxins, and thiol-dependent systems neutralize reactive species and maintain redox balance. These defense mechanisms not only enhance bacterial survival during infection but also contribute to virulence, persistence, and resistance to host immune clearance (Staerck et al., 2017).

The interaction between host and bacterial redox responses creates an oxidative environment that may also influence antimicrobial activity. In the case of colistin, its primary bactericidal mechanism involves disruption of the Gram-negative outer membrane; however, oxidative stress has been proposed as an additional contributor to its antibacterial effects. Experimental studies have reported increased ROS generation after exposure to polymyxins, suggesting that disruption of bacterial redox homeostasis may participate in cell death (Yu et al., 2017). Nevertheless, whether ROS production is a causal determinant of colistin lethality or a secondary consequence of membrane disruption and downstream cellular dysfunction remains controversial (Trimble et al., 2016).

This issue is part of a broader debate regarding ROS-dependent antibiotic killing. Kohanski et al. (2007) proposed that bactericidal antibiotics may converge on a common oxidative pathway involving perturbations in central metabolism, disruption of iron–sulfur clusters, Fenton chemistry, and increased hydroxyl radical formation. However, this general hypothesis was later challenged by studies demonstrating that quinolones, β-lactams, and aminoglycosides can retain bactericidal activity under conditions that restrict ROS formation. These studies also failed to consistently detect biochemical hallmarks of oxidative damage, thereby questioning the requirement for ROS as a universal mechanism of antibiotic-induced bacterial death (Keren et al., 2013). Importantly, as polymyxins were not included in these analyses, their conclusions cannot be directly extrapolated to colistin.

For polymyxins specifically, experimental evidence supports an association between colistin exposure and oxidative stress; however, the contribution of ROS to bacterial killing remains incompletely defined (Trimble et al., 2016; Yu et al., 2017). Thus, rather than representing an established primary mechanism of colistin action, redox disruption may function as a secondary or complementary component of its antibacterial activity. This uncertainty is particularly relevant in the broader context of colistin therapy, where redox alterations are also strongly implicated in drug-induced host toxicity.

## 4 Redox mechanisms of colistin-induced toxicity

Colistin-induced nephrotoxicity is a multifactorial process that begins with drug accumulation in proximal tubular epithelial cells and progresses through interconnected mechanisms involving mitochondrial dysfunction, oxidative stress, inflammation, endoplasmic reticulum stress, and regulated cell death. Acute kidney injury commonly appears within the first week of therapy, with risk increasing according to cumulative dose and treatment duration. Rather than resulting from a single pathway, colistin toxicity reflects coordinated cellular stress responses, including activation of apoptotic signaling through Bcl-2/Bax, Fas/FasL, CHOP, and caspase-12 pathways. Within this framework, redox dysregulation emerges as a central mechanism linking mitochondrial injury, inflammatory activation, and tubular damage (Diani et al., 2024; Mahmoud and Alharbi, 2025).

Mechanistic studies indicate that colistin directly disrupts mitochondrial membrane potential, supporting mitochondria as an early intracellular target rather than a downstream consequence of cellular injury. This places mitochondrial dysfunction at a central point in colistin-induced nephrotoxicity and links intracellular drug accumulation to oxidative damage (Azad et al., 2019; Gai et al., 2019).

Mitochondrial dysfunction in colistin-induced nephrotoxicity is closely associated with broader disruption of redox homeostasis, extending beyond excessive ROS generation. Preclinical evidence shows that colistin impairs antioxidant defenses and alters pathways related to energy metabolism, inflammation, and apoptosis, indicating that oxidative stress is part of an integrated network of cellular injury rather than an isolated event. Moreover, damaged proximal tubular epithelial cells actively amplify renal inflammation by inducing mediators such as IL-34, which promote macrophage recruitment and further contribute to tissue damage (Azad et al., 2019; Gai et al., 2019; Mahmoud and Alharbi, 2025). Transcriptomic analyses have also demonstrated alterations in the expression of genes associated with oxidative stress, apoptosis, DNA replication, and cell-cycle regulation, indicating that colistin-induced nephrotoxicity involves coordinated molecular responses beyond oxidative injury alone (Romero-Durán et al., 2024).

Although redox alterations are implicated in both the antibacterial activity and host toxicity of colistin, these processes are not mechanistically equivalent. In bacteria, oxidative stress has been proposed as a secondary or complementary contributor to colistin-mediated killing, although its causal role remains controversial. In renal proximal tubular epithelial cells, by contrast, redox dysregulation is more consistently linked to mitochondrial dysfunction, impaired antioxidant defenses, inflammatory signaling, and cellular injury. Thus, the antibacterial and toxic effects of colistin may converge at the level of redox imbalance, yet they diverge in their initiating events, cellular context, downstream pathways, and biological consequences. This distinction is particularly relevant for the development of redox-based adjuvant strategies, which should ideally attenuate host oxidative injury without compromising the antibacterial activity of colistin.

## 5 Redox modulation as an adjuvant therapeutic strategy

### 5.1 Preclinical evidence

The identification of oxidative stress as a central contributor to colistin-induced nephrotoxicity has encouraged the development of host-directed strategies aimed at preserving redox homeostasis during therapy. Since discontinuing colistin may compromise treatment of life-threatening multidrug-resistant Gram-negative infections, adjunctive compounds have been investigated to reduce renal injury while maintaining antibacterial efficacy (Nation et al., 2019; Romero-Durán et al., 2024).

Preclinical research consistently demonstrates that antioxidant interventions mitigate colistin-induced renal injury. Across these models, antioxidant compounds attenuate tubular damage, improve renal histopathology, preserve endogenous defense systems, decrease lipid peroxidation, and reduce apoptotic signaling. Their nephroprotective profile is further supported by improvements in conventional renal function markers, including serum creatinine and blood urea nitrogen, as well as emerging biomarkers such as KIM-1, NGAL, NAG, and cystatin C. Overall, this body of evidence supports redox homeostasis preservation as a promising strategy to reduce the severity of colistin-induced nephrotoxicity (Mirjalili et al., 2022; Nation et al., 2019).

### 5.2 Clinical evidence

Although antioxidant interventions demonstrate robust and reproducible nephroprotective activity in preclinical models, their clinical applicability remains limited and heterogeneous (Brubaker and Lauffenburger, 2020; Mosayebi et al., 2022; Samsami et al., 2021). To date, clinical evidence is restricted to a small number of studies evaluating agents such as ascorbic acid, N-acetylcysteine (NAC), and vitamin E, most of which have yielded inconclusive results (Table 1). The gap between robust preclinical efficacy and uncertain clinical benefit reflects both methodological and biological complexity. Most clinical studies have enrolled small and heterogeneous patient populations, reducing statistical power and limiting generalizability. Antioxidant regimens vary widely in dose, duration, route, and timing of administration, as well as in the criteria used to define acute kidney injury, making direct comparisons difficult (Al Sulaiman et al., 2022; Brubaker and Lauffenburger, 2020; Dalfino et al., 2015; Marshall et al., 2023; Mosayebi et al., 2022; Radabaugh et al., 2023; Samsami et al., 2021).

**TABLE 1:** Summary of clinical trials evaluating antioxidant interventions for the prevention of colistin-induced acute kidney injury (AKI).

Agent	*n* (sample size)	Dose/regimen	AKI outcome	Key limitation
Ascorbic acid (Dalfino et al., 2015)	70	3 g IV every 12 h	Sole independent protective factor (aHR 0.27; *p* < 0.001)	Prospective observational design (not an RCT).
Ascorbic acid (Al Sulaiman et al., 2022)	90	1,000 mg/day enterally (median 3–6 days)	No significant difference (44.4% vs. 40.0%; *p* = 0.68)	Retrospective design; potentially insufficient dose/route.
N-acetylcysteine (Mosayebi et al., 2022)	82 (43 NAC, 39 control)	600 mg twice daily for 10 days	Numerically lower, but not significant (18.6% vs. 28.2%; *p* = 0.303)	Open-label design; small sample size.
Vitamin E (Samsami et al., 2021)	52 (26 per group)	400 IU/day orally or via nasogastric tube	No significant difference (42.3% vs. 46.2%; *p* = 0.78)	Small sample size; oral/NG administration route.

AKI, acute kidney injury; aHR, adjusted hazard ratio; ICU, intensive care unit; IV, intravenous; NAC, N-acetylcysteine; NG, nasogastric; RCT, randomized controlled trial.

Pharmacokinetic (PK) optimization offers a non-redox approach to balance efficacy and toxicity through loading doses, renal-adjusted maintenance, and therapeutic drug monitoring. However, colistin’s narrow therapeutic window and substantial interindividual PK variability complicate this approach, as therapeutic plasma exposures inherently overlap with nephrotoxic ranges. Because PK-guided dosing cannot directly target the cellular mechanisms underlying renal injury, complementary host-directed strategies remain essential to mitigate nephrotoxicity without compromising antibacterial efficacy (Nation et al., 2019; Tsuji et al., 2019).

Preserving redox homeostasis remains a biologically plausible therapeutic strategy; however, its clinical value requires confirmation in adequately powered randomized controlled trials using standardized treatment protocols, validated kidney injury biomarkers, and clinically meaningful outcome measures.

### 5.3 Translational challenges

The mismatch between the strong nephroprotective effects observed in preclinical models and the limited benefit seen in patients highlights a central challenge in translational pharmacology. Although antioxidant interventions attenuate colistin-induced nephrotoxicity under controlled experimental conditions, these effects have not been reliably reproduced in clinical settings. This limitation extends beyond colistin and reflects a broader difficulty in developing adjunctive therapies that target oxidative stress, where promising preclinical strategies often fail to achieve meaningful clinical efficacy (Gao et al., 2025; Li et al., 2025; Mitchell et al., 2025).

From a translational research perspective, the limited clinical benefit likely reflects a mismatch between simplified preclinical models and the biological complexity of patients treated with colistin. Preclinical studies typically use young, healthy animals exposed to standardized colistin regimens under tightly controlled conditions. In contrast, patients receiving colistin are often critically ill, with advanced age, multiple comorbidities, sepsis, systemic inflammation, and exposure to concomitant nephrotoxic medications. These factors can substantially alter mitochondrial function, redox homeostasis, inflammatory responses, and renal susceptibility to injury, creating a biological environment that is far more heterogeneous than that reproduced in conventional animal models (Gao et al., 2025; Li et al., 2025; Mitchell et al., 2025).

Clinical translation is also limited by methodological heterogeneity across studies. Variation in antioxidant selection, dosing strategy, treatment duration, timing of administration relative to colistin exposure, and diagnostic criteria for acute kidney injury limits comparability and may mask beneficial effects in specific patient subgroups. This is particularly relevant because colistin-induced renal injury is not driven by oxidative stress alone, but by an interconnected network of mitochondrial dysfunction, inflammation, vascular alterations, and regulated cell death. Accordingly, interventions aimed only at reducing oxidative stress may offer limited protection if initiated after these pathogenic processes are already established (Al Sulaiman et al., 2022; Brubaker and Lauffenburger, 2020; Gao et al., 2025; Li et al., 2025; Marshall et al., 2023; Mitchell et al., 2025; Radabaugh et al., 2023).

Improving the predictive value of preclinical research will require models that more closely reproduce the clinical setting. The incorporation of aged animals, disease-specific models, clinically relevant dosing regimens, and polypharmacy protocols may better capture the heterogeneity of patients treated with last-resort antibiotics. Emerging technologies, including kidney organoids and organ-on-chip platforms, offer additional opportunities to evaluate drug-induced nephrotoxicity in human-relevant systems while supporting mechanistic studies of redox biology. In parallel, artificial intelligence and machine learning approaches may help integrate multidimensional datasets from preclinical models, biomarkers, and clinical cohorts to identify susceptibility profiles and improve prediction of nephrotoxicity risk. Together, these approaches may help bridge the current gap between experimental nephroprotection and clinical efficacy by improving the translational relevance of preclinical models (Gao et al., 2025; Garcia-Llorens et al., 2025; Li et al., 2025; Silverstein et al., 2026).

## 6 Future perspectives

Advances in the understanding of redox biology in colistin-induced nephrotoxicity support a shift from empirical supportive care toward mechanism-based therapeutic strategies. Future research should move beyond the identification of new antioxidant compounds and address which patients are most likely to benefit from redox-targeted interventions. Given the substantial interindividual variability in oxidative stress responses, biomarker-guided patient stratification should become a central element of future clinical trial design and therapeutic decision-making (Silverstein et al., 2026).

A further priority is the development and validation of sensitive biomarkers capable of detecting kidney injury before irreversible functional decline occurs. Although serum creatinine and blood urea nitrogen remain widely used indicators of renal function, both have limited sensitivity during the early stages of nephrotoxicity and are influenced by multiple non-renal factors. Future studies should therefore evaluate multimarker approaches integrating complementary dimensions of renal injury, such as functional markers (e.g., serum creatinine and cystatin C), tubular injury biomarkers (e.g., KIM-1, NGAL, and NAG), together with indicators of oxidative stress, mitochondrial dysfunction, and inflammation. Rather than relying on individual biomarkers, such panels could potentially improve early detection, toxicity classification, therapeutic monitoring, and identification of patients at increased risk of colistin-induced nephrotoxicity. However, their predictive performance and clinical utility require prospective validation before they can be incorporated into therapeutic decision-making (Ahamad et al., 2026; Nation et al., 2019; Piko et al., 2023; Romero-Durán et al., 2024).

Mitochondria represent a relevant target for next-generation host-directed interventions. Because mitochondrial impairment connects oxidative stress, inflammation, and tubular cell death, strategies aimed at preserving mitochondrial integrity may offer advantages over conventional antioxidant supplementation. Mitochondria-targeted antioxidants, such as MitoQ, and emerging delivery systems designed to accumulate selectively within damaged mitochondria may enhance intracellular bioavailability while limiting interference with physiological redox signaling. Although these approaches remain largely experimental, they provide a rational framework for reducing nephrotoxicity without compromising the antibacterial activity of colistin (Franczyk et al., 2026; Romero-Durán et al., 2024).

Recent advances in nanotechnology, systems biology, and computational approaches further expand the therapeutic landscape of redox modulation. Redox-responsive nanomaterials capable of selectively releasing antioxidants within oxidative microenvironments may overcome several limitations associated with conventional antioxidant therapy, including poor bioavailability and limited tissue specificity. In parallel, artificial intelligence and machine learning may facilitate the integration of multidimensional datasets, including biomarker profiles, omics data, clinical variables, and preclinical readouts, to identify redox phenotypes and develop predictive models of nephrotoxicity risk. Together, these technologies may support a more precise translation of redox biology into clinically applicable strategies for the management of antibiotic-induced toxicity (Ahamad et al., 2026; Garcia-Llorens et al., 2025; Nahar and Sohag, 2025).

The success of redox-based interventions will ultimately depend on selectively limiting pathological oxidative injury without interfering with physiological redox signaling or compromising antibacterial activity. Future strategies should therefore integrate mechanistically targeted host protection with optimized colistin therapy and prospective clinical validation. Achieving this balance will be essential for translating redox biology into clinically meaningful approaches that reduce nephrotoxicity while preserving antimicrobial efficacy (Ahamad et al., 2026; Garcia-Llorens et al., 2025).

## 7 Conclusion

Redox biology plays a central role in both the antibacterial activity and host toxicity of colistin. While redox alterations may contribute to bacterial killing, excessive oxidative activation in renal tissues promotes mitochondrial dysfunction, inflammation, and tubular cell death, limiting its clinical use. Although preclinical evidence supports redox modulation as a promising strategy to reduce colistin-induced nephrotoxicity, clinical translation remains limited. Future progress will depend on deeper mechanistic understanding, biomarker-guided patient selection, and targeted interventions that protect organ function without compromising antibacterial efficacy. These advances may enable safer and more effective use of last-resort antibiotics in the context of antimicrobial resistance.
